# Individual values and spirituality and their meaning for affective well-being and engagement with life in very old age

**DOI:** 10.1007/s00391-021-01974-9

**Published:** 2021-10-01

**Authors:** Marcella Reissmann, Anna Storms, Christiane Woopen

**Affiliations:** 1 Cologne Center for Ethics, Rights, Economics, and Social Sciences of Health, University of Cologne, Albertus Magnus Platz, 50923 Cologne, Germany; 2 a.r.t.e.s. Graduate School for the Humanities Cologne, University of Cologne, Cologne, Germany; 3 Research Unit Ethics, Medical Faculty, University Hospital Cologne, Cologne, Germany

**Keywords:** Quality of life, Old age, Representative survey, Growth, Connectedness, Lebensqualität, Hochaltrigkeit, Repräsentative Studie, Wachstum, Verbundenheit

## Abstract

**Background:**

Individuals’ ideals and aspirations are considered to constitute a central reference frame for subjective evaluations of their perceived reality, and, thus, to be crucial for individual quality of life (QoL) outcomes. By examining individual values and spirituality in very old people, the aim of this study was to describe two constructs representing the aspirations of the individual, as well as the relation of these constructs to both hedonic and eudaimonic QoL outcomes in very old age (VOA).

**Material and methods:**

Cross-sectional data from a representative survey of people in VOA (NRW80+, *n* = 1863) were used. Individual values were assessed based on the Portrait Value Questionnaire. A revised questionnaire was developed drawing on the Spiritual Health and Life-Orientation Measure. Individual values and spirituality were studied using descriptive statistics, and hierarchical linear regression models were used to analyze their predictive value for two QoL outcomes: 1) affective well-being as an indicator of hedonic QoL, which was assessed using the positive affect subscale of the short form of the Positive and Negative Affect Schedule, and 2) engagement with life, which captures eudaimonic aspects and which was measured with a subscale of the Valuation of Life Scale.

**Results:**

The most important values were both protection and growth-oriented values with a social focus. However, only values representing strivings for growth had a positive association with QoL outcomes. Spirituality was of high relevance to very old people, although not in the sense of religious institutions or practices. Rather, it predominantly consisted in environmental, interpersonal, and transcendental connectedness, all of which were positively connected to QoL outcomes.

**Conclusion:**

Individual values and spirituality can be an important resource for hedonic as well as eudaimonic QoL; however, age-related losses may lead to an emphasis of protective values that are not beneficial in terms of QoL. To support older people on their spiritual journey, a broad concept of spirituality needs to be established among researchers as well as practitioners.

**Supplementary Information:**

The online version of this article (10.1007/s00391-021-01974-9) contains supplementary material, which is available to authorized users.

## Abstract (de)

**Hintergrund:**

Es wird angenommen, dass Individuen ihre Realität auf Basis ihrer persönlichen Ideale und Bestrebungen bewerten. Diese sind demnach äußerst wichtig für individuelle Lebensqualitätsresultate (LQR). Die vorliegende Studie fokussierte die individuellen Werte und Spiritualität hochaltriger Menschen. Damit sollten zwei Konstrukte, die die Bestrebungen Hochaltriger (HA) repräsentieren, sowie ihr Zusammenhang mit hedonischen und eudaimonischen LQR in der Hochaltrigkeit untersucht werden.

**Material und Methoden:**

Es wurden Querschnittsdaten einer repräsentativen Befragung von HA (NRW80+, *n* = 1863) genutzt. Individuelle Werte wurden angelehnt an den Portrait Value Questionnaire operationalisiert. Zur Erhebung von Spiritualität wurde ein auf Basis des Spiritual Health and Life-Orientation Measure entwickelter Fragebogen eingesetzt. Individuelle Werte und Spiritualität wurden zunächst deskriptiv analysiert. Mittels hierarchischer linearer Regressionsmodelle wurde ihre Vorhersagekraft für zwei LQR untersucht: 1) affektives Wohlbefinden als Indikator hedonischer LQ, welches mit der Subskala positiver Affekt der Kurzform der Positive and Negative Affect Schedule erhoben wurde, 2) aktive Verbundenheit mit dem Leben, welche Aspekte eudaimonischer LQ abbildet und mit der Subskala Engagement der Valuation of Life Scale gemessen wurde.

**Ergebnisse:**

Die für HA wichtigsten individuellen Werte waren sowohl protektive als auch wachstumsorientierte Werte mit sozialem Fokus. Allerdings waren nur wachstumsorientierte Werte positiv mit LQR assoziiert. Spiritualität zeigte eine hohe Relevanz für HA, jedoch nicht im Sinne religiöser Institutionen oder Praktiken. Vielmehr bestand sie in Umwelt-, interpersoneller und transzendentaler Verbundenheit, welche außerdem positiv zu LQR beitrugen.

**Diskussion:**

Individuelle Werte und Spiritualität können eine wichtige Ressource für hedonische wie auch eudaimonische LQR sein. Verluste im Alter könnten jedoch zu einer Betonung protektiver Werte führen, welche nicht zu positiven LQR beitragen. Ein breites Verständnis von Spiritualität sollte sowohl in der Forschung als auch in der Praxis etabliert werden, um HA in ihrer spirituellen Entwicklung zu unterstützen.

## Background

According to models of cognitive discrepancy, value-based preferences as a central reference frame for subjective evaluations of actual living conditions are crucial to understand quality of life (QoL) outcomes [29, 30]. Extending previous perspectives on QoL, the challenges and potentials (CHAPO) model of QoL of the very old [43] thus considers the system of individual goals and values an important resource for QoL outcomes. Addressing QoL outcomes beyond the hedonic realm, the model also includes eudaimonic conceptions of QoL. These are expected to be of particular relevance in very old age as older adults face significant challenges in maintaining a sense of purpose in life [13, 20, 34]. By considering individual values and spirituality, this article describes two constructs representing the standards of the individual and examines their relation to both hedonic and eudaimonic QoL outcomes in very old age (VOA; i.e. 80 years and above).

### Individual values.

**Fig. 1:**
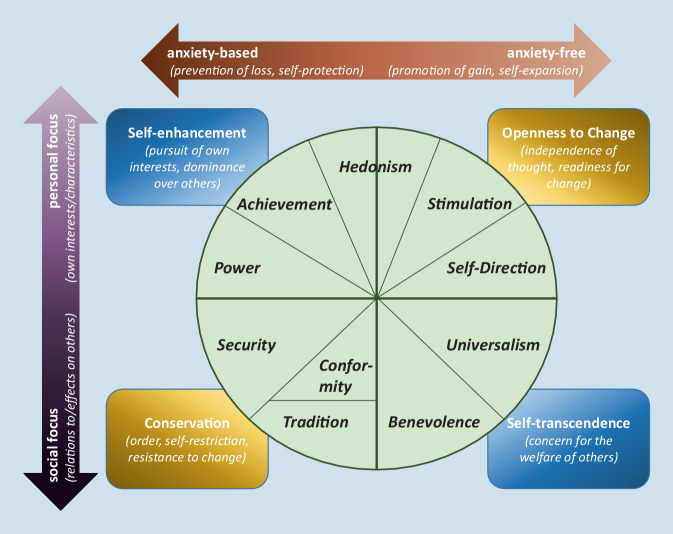
Model of value relations and contents. Own illustration summarizing figures and information by Schwartz [38]. The closer any two values are located to each other within the circle, the more congruent—the more distant, the more conflicting their underlying motivations

The values held by individuals determine what is important to them in life [38]. In his theory of basic human values, Schwartz [36, 38] identified ten basic values expressing distinct goals that motivate action. To illustrate the relations among values, he arranged them in a circular model split by two higher order dimensions (see Fig. 1). The first dimension contrasts ‘openness to change’ with ‘conservation’ values: Values belonging to the first type aim at independence of thought and action, as well as readiness for change, whereas values of the latter type are directed at order, self-restriction, and are resistant to change. The second dimension represents a conflict between ‘self-enhancement’ values, which emphasize the pursuit of one’s own interests and dominance, and ‘self-transcendence’ values, which emphasize the concern for the welfare and success of others. The contents of the ten values can be further described by which interests they serve: While some regulate the expression of one’s own interests (personal focus), others focus on one’s relation to and influence on others (social focus). Furthermore, values can be distinguished by their relation to anxiety: Some values strive for self-protection (anxiety-based), whereas others strive for growth or self-expansion (anxiety-free).

According to Schwartz’s theory, individuals ascribe a certain importance to each value in relation to the other values. For instance, an individual striving for power will ascribe high importance also to its neighboring values within the value circle, but low importance to opposite values. The resulting individual profile of value priorities guides action and serves as a transsituational reference system for the evaluation of one’s reality.

Empirical research has examined the relations of individual values with well-being both in student samples and in representative samples from the European Social Survey. Studies reported a tendency of power values being negatively related to well-being, and benevolence, stimulation, self-direction, and hedonism values being positively related to well-being (for an overview, see [39]); however, findings are not completely consistent, and they revealed differences across social contexts (e.g. [40]).

Besides social contexts, there is reason to suggest age-related differences in the relevance of individual values and their relations to well-being. Due to the tipping of the balance between gains and losses that develops in later life, life span theories predict that older persons would place higher importance on goals focusing on loss prevention (i.e., anxiety-based values) instead of growth (i.e., anxiety-based values) [7]. Furthermore, a decreased self-centeredness and a stronger focus on the welfare of others can be expected based on Erikson’s stage theory of human development [8]. This is also in line with the model of gero-transcendence [41] and its idea that older people focus more strongly on social and emotional values than on material values. In line with this, empirical research including studies drawing on large multinational datasets (European Social Survey, World Values Survey) has observed an age-related shift from self-enhancement to self-transcendence and from openness to change to conservation values (e.g., [5, 33, 42]), even across individualistic and collectivistic cultures [12]. Against this theoretical and empirical background, one could expect that people in VOA clearly prioritize and benefit from socially focused values. On the other hand, psychological threat is expected to increase the priority people give to micro-worries (problems close to the self) compared to macro-worries (problems external to the self) [4]. This has been confirmed empirically in a sample of palliative care patients [9]. In the light of age-related changes and losses, thus, one could claim a returning rise of values with personal focus in VOA. So far, it has not yet been investigated which values are prevalent in VOA and how they relate to QoL outcomes in this particular phase of life.

### Spirituality.

Another interpretative framework for the finding of purpose in life [19] and the “understanding [of] the concepts of self and world during the course of one’s life” [17, p. 2] is spirituality. It is not necessarily linked to religion but involves a belief in something bigger than oneself. Spirituality can consist in a feeling of connectedness with other human beings (interpersonal connectedness), the non-human environment (environmental connectedness), or with God or a higher power (transcendental connectedness) [10, 19]. Additionally, European qualitative research showed that spirituality, especially in the understanding of older people, comprises a religious conduct of life (vita spiritualis), which finds its expression in participation in communities of faith or religious practices ([19], cf. [32]); however, although people in old age show a stronger traditional religiousness compared to younger generations [32], there are also hints on decreasing formal religiousness and increasing private spirituality in older age: While functional impairments impede participation in formal religious practices (e.g., church attendance), they do not constrain existential preoccupation with the self and the world [19].

Numerous studies have reported a positive relation of spirituality/religiosity with well-being, sense in life, and aims in life [19, 28]. Several models of health consider spirituality a protective factor and a means of resilience to cope with diseases and critical life events [26], which has become a main focus of quantitative research on the functions of spirituality. Especially in end-of-life care, spirituality is therefore implemented as an independent dimension besides physical, psychological, and social health [44]. As a source of subjective interpretation of the self and the world offering a sense of personal meaning, control beyond one’s own resources, and comfort [25], spirituality might be an important resource to encounter the challenges and existential questions arising in VOA; however, research on spirituality mostly focuses on its role for specific subgroups (e.g., the chronically or terminally ill). Furthermore, most instruments ignore the multifaceted nature of spirituality [26] and do not clearly distinguish spirituality from religiosity, often rather assessing religiosity than spirituality [19]. Moreover, most studies on spirituality have been conducted in America. Knowledge about the understanding and role of spirituality in the very old population of Germany therefore remains limited.

### Objectives.

Against the background of the described desiderata, the present study makes an explorative contribution to the knowledge about individual values and spirituality in VOA. It shall explore which individual values, and which of the different domains of spirituality are particularly relevant in VOA, and how they are related to two QoL outcomes: 1) affective well-being as an indicator of hedonic QoL, and 2) engagement with life, which captures eudaimonic aspects in the sense of “purpose, meaningfulness, persistence, and self-efficacy” [27, p. 407].

## Material and methods

### Sampling

The present study used data from the survey “Quality of Life and Well-Being of the Very Old in North Rhine-Westphalia (NRW80+)”. The sample is representative of the population aged 80 years and older in Germany’s most populated federal state. Both community-dwelling and institutionalized individuals are covered. Men and those belonging to older age groups (85–89 years, 90+ years) were oversampled to allow for valid cross-group comparisons. For more information regarding the study and sample, see [14]. The total of 1863 interviews includes 176 interviews with proxy informants that were conducted when target persons could not be interviewed themselves due to severe health impairments. Information on the use of proxy information for the analyses presented here can be found in the electronic supplement (appendix A).

### Measures

#### Independent variables

##### Individual values.

Individual values were assessed based on a 10-item version of the portrait value questionnaire (PVQ, [6, 37]). The original PVQ asks respondents to compare themselves with portraits of people with different aspirations. As a feasibility study prior to NRW80+ [22] showed that this concept was nonviable in a sample of very old respondents, items were modified into direct questions, e.g. “How important is it to you to do things your own way?” (self-determination). Answers were given on a 4-point Likert scale (1 = not important at all, 4 = very important). Initial psychometric analysis of the scale found that in our data the relationships between the 10 values could best be represented by an exploratory structural equations [1] three factor model. Conformity, security and stimulation (inverted) are markers of “conservation vs. openness to change”. “Self-enhancement” is represented by power, achievement, hedonism and stimulation, whereas key markers of “self-transcendence” are universalism, benevolence, self-direction, and tradition. Although theoretically suggested, self-transcendence and self-enhancement do not appear to be opposite ends of a continuum but to be moderately related to each other (r = 0.69) in the current sample. Overall, the model did not exactly match Schwartz’s theory, indicating that the values have a partly different meaning to very old adults than to younger groups. This led us to use the 10 values as single items for this initial analysis of individual values of people in VOA.

##### Spirituality.

Based on the established spiritual health and life-orientation measure [10], a shorter and revised questionnaire on spirituality (QueSt) was developed. Here, seven items referring to the importance of the above described elements of spirituality were used (e.g., for transcendental connectedness: “How important is it to you to feel connected to God or a higher power?”). Importance was rated on a 4-point response scale (1 = not important at all, 4 = very important).

##### Control variables.

Control variables are listed in Table 1. Information on their measurement is provided in the electronic supplement (appendix B).

**Table 1:** Descriptive statistics of control variables, individual values, spirituality, and QoL outcomes (*n* = 1863)

		M (SD)/%
Age		85.14 (4.20)
Gender	Female	63.7%
	Male	36.3%
Education	Low	30.6%
	Middle	51.2%
	High	18.2%
Multimorbidity		0.18 (0.12)
Full in-patient care		13.0%
*Individual values*		
1. Security		3.61 (0.67)
2. Self-direction		3.46 (0.70)
3. Tradition		3.30 (0.88)
4. Universalism		3.20 (0.90)
5. Hedonism		2.97 (0.89)
6. Conformity		2.94 (1.03)
7. Benevolence		2.76 (0.96)
8. Achievement		2.60 (0.94)
9. Power		1.94 (0.88)
10. Stimulation		1.57 (0.80)
*Spirituality*		
Transcendental connectedness		2.91 (1.05)
Interpersonal connectedness		3.44 (0.69)
Environmental connectedness		3.47 (0.73)
Institutionalized religion		2.39 (1.17)
Feeling part of a greater whole		2.56 (1.04)
Religious practices		2.61 (1.20)
Faith or spirituality in life		2.80 (1.11)
*QoL outcomes*		
Affective well-being		3.25 (0.89)
Engagement with life		1.45 (0.62)

Weighted data. Results obtained from imputed dataset. App. C in the electronic supplement shows descriptive statistics based on the original dataset. Value ranges: for multimorbidity: 0 to 1; for individual values and spirituality: 1 (not important at all) to 4 (very important); for AWB: 1 (never) to 5 (very often); for EwL: 0 (no) to 2 (yes)

#### Dependent variables

##### *Affective well-being (AWB)*.

The frequency of positive affective states (e.g. “elated”) experienced during the past 12 months was measured with the positive affect subscale of the short form of the positive and negative affect schedule [24]. Answers were given on a 5-point Likert scale (1 = never, 5 = very often). Internal consistency test showed high reliability of the scale (Cronbach’s α = 0.89). The mean of all answers was used as an indicator of AWB.

##### Engagement with life (EwL).

The two-factor structure [13] of the Valuation of Life Scale [27] was replicated in the current sample. Response options were reduced to a 3-point scale (0 = no, 1 = neither/nor, 2 = yes) as has been recommended for samples of very old persons [20]. As an indicator of EwL, the total mean of the subscale engagement with five items (e.g. “Do you feel able to accomplish your life goals?”) was used. The EwL subscale showed high reliability (Cronbach’s α = 0.87).

### Statistical analyses

After descriptive analyses of all variables, hierarchical linear regression models were conducted to examine the predictive potential of individual values and spirituality for AWB and EwL. All analyses used weighted data to correct for the disproportional age and gender distribution as well as nonresponse in the sample and were conducted using SPSS software version 27 (IBM Corp., Armonk, NY, USA).

#### Multiple imputation.

One item on QueSt (importance of feeling part of a greater whole) had a large number of missing values (17.6%). Five more variables exceeded 4% of missing values: educational background (4.5%), as well as individual values—hedonism (4.1%), stimulation (4.2%), achievement (4.5%), and benevolence (6.2%). In all other variables, the share of missing values varied between 0% and 3.9% (median = 2.7). The number of missing values cumulated in a reduction of the final analysis sample by 32.5%. Therefore, the multiple imputation function within SPSS 27 was used. All variables described above plus a seven-tier measure of monthly income were used to run five imputations using a linear procedure.

## Results

Table 1 summarizes the descriptive characteristics of the very old population of NRW. Security was the most important value, followed by self-direction, tradition, and universalism. As the observed hierarchy of value priorities indicated a generally higher relevance of values with social focus compared to values with higher personal focus, we decided to conduct further analyses at this point. For this purpose, we aggregated the five values with higher social focus and higher personal focus. They showed acceptable internal consistency (social focus: Cronbach’s alpha = 0.61; personal focus: Cronbach’s alpha = 0.51). A paired-samples t‑test confirmed a very large difference between means on values with a higher social focus (M = 3.2, SD = 0.6) and those with a higher personal focus (M = 2.5, SD = 0.5; t(1862) = −48.132; *p* < 0.001; d = 1.14).

Regarding spirituality, environmental connectedness was most important to the very old population, followed by interpersonal and transcendental connectedness. Religious institutions and practices were of subordinate but still moderate importance. With respect to QoL outcomes, very old persons showed a moderate AWB, but a rather high EwL.

Table 2 shows the results retrieved from regression models. Tolerance values for the included variables varied between 0.29 and 0.92, showing no indication of multicollinearity. The final model predicting AWB explained 32.1% of variance in AWB. A positive association with AWB was found for environmental and interpersonal connectedness, self-direction, benevolence, universalism, hedonism, and stimulation, whereas conformity and power related negatively with AWB.

**Table 2:** Hierarchical regression models predicting affective well-being and engagement with life by spirituality and individual values (*n* = 1863)

	AWB (I)	AWB (II)	AWB (III)	EwL (I)	EwL (II)	EwL (III)
	*β *(SE)^a^	*β *(SE)^a^	*β *(SE)^a^	*β *(SE)^a^	*β *(SE)^a^	*β *(SE)^a^
Constant	4.9 (. 47)***	3.164 (0.17)***	1.517 (0.45)***	2.863 (0.31)***	1.606 (0.30)***	0.338 (0.02)
*Control variables*						
Age	−0.08 (0.01)***	−0.07 (0.01)**	−0.03 (0.01)	−0.03 (0.00)***	−0.07 (0.00)***	−0.03 (0.00)
Gender (ref: male)	0.10 (0.05)***	0.07 (0.05)**	0.06 (0.04)*	−0.03 (0.03)	−0.05 (0.03)*	−0.06 (0.03)**
Low education (ref: high)	−0.20 (0.07)***	−0.14 (0.06)***	−0.09 (0.06)**	−0.11 (0.04)***	−0.07 (0.04)*	−0.02 (0.04)
Medium education (ref: high)	−0.12 (0.06)***	−0.09 (0.06)**	−0.07 (0.05)*	−0.05 (0.04)	−0.02 (0.04)	−0.01 (0.03)
Full in-patient care	−0.20 (0.07)***	−0.12 (0.07)***	−0.08 (0.06)***	−0.34 (0.04)***	−0.25 (0.04)***	−0.22 (0.04)***
Multimorbidity	−0.10 (0.17)***	−0.10 (0.16)***	−0.10 (0.15)***	−0.13 (0.11)***	−0.14 (0.10)***	−0.13 (0.10)***
*Spirituality*						
Transcendental connectedness	–	−0.01 (0.03)	−0.01 (0.03)	–	0.05 (0.02)	0.05 (0.02)
Interpersonal connectedness	–	0.13 (0.03)***	0.06 (0.03)**	–	0.11 (0.02)***	0.05 (0.02)*
Environmental connectedness	–	0.21 (0.03)***	0.08 (0.03)**	–	0.23 (0.02)***	0.09 (0.02)***
Institutionalized religion	–	0.04 (0.03)	0.03 (0.02)	–	0.07 (0.02)*	0.07 (0.02)*
Part of a greater whole	–	0.12 (0.03)***	0.05 (0.03)	–	0.10 (0.02)**	0.04 (0.02)
Religious practices	–	−0.04 (0.03)	−0.04 (0.03)	–	−0.12 (0.02)**	−0.12 (0.02)***
Faith or spirituality in life	–	−0.02 (0.03)	−0.02 (0.03)	–	−0.03 (0.02)	−0.02 (0.02)
*Individual values*						
Self-direction	–	–	0.23 (0.03)***	–	–	0.21 (0.02)***
Power	–	–	−0.07 (0.02)**	–	–	−0.02 (0.01)
Security	–	–	−0.03 (0.03)	–	–	−0.02 (0.02)
Hedonism	–	–	0.09 (0.02)***	–	–	0.06 (0.02)**
Benevolence	–	–	0.16 (0.03)***	–	–	0.11 (0.02)***
Achievement	–	–	0.04 (0.02)	–	–	0.08 (0.01)***
Stimulation	–	–	0.08 (0.03)***	–	–	0.07 (0.02)***
Conformity	–	–	−0.11 (0.02)***	–	–	−0.00 (0.01)
Universalism	–	–	0.09 (0.03)***	–	–	0.14 (0.02)***
Tradition	–	–	0.02 (0.03)	–	–	−0.03 (0.02)
*R*_*adj*_^*2b*^	0.088***	0.183***	0.321***	0.172***	0.270***	0.384***

Weighted data. Results obtained from imputed dataset. App. D in the electronic supplement provides information on differing results obtained from the original dataset

*p ≤ 0.05 **p ≤ 0.01 ***p ≤ 0.001

^a^Coefficients and standard errors calculated as mean values from all imputed data sets as SPSS does not support calculation of values for the combined dataset

^b^Calculated as the mean value of *Radj*^2^ in all imputed data sets

Regarding EwL, 38.4% of its variance could be explained with the final model. Environmental and interpersonal connectedness as well as institutionalized religion had a positive association with EwL, while high importance of religious practices predicted lower EwL. Self-direction, universalism, benevolence, achievement, stimulation, and hedonism were positively related to EwL. None of the values related negatively to EwL on a significant level.

## Discussion

In our representative data of very old persons, the most important individual values belonged almost exclusively to the dimensions of conservation and self-transcendence, both of which focus on social relationships and one’s influence on others rather than on personal interests [38]. Additional analyses confirmed significantly higher means on values with a higher social focus than on those with a higher personal focus. Taken together, our findings support assumptions of a decreased self-centeredness and increased concern for the welfare of others in old age [8, 41] and are consistent with previous research on value priorities in later life (e.g. [5, 33, 42]), replicating it for VOA.

However, the second most important value found in our study was self-direction, which represents strivings for personal growth [38]. One could argue that in VOA, living and acting self-determinedly is less self-evident, no longer necessarily representing growth but evolving into a state that needs to be protected from the threat of depending on others, for example due to health restrictions. Borg et al. [5] have identified age-associated differences in the understanding of and relations between values, i.e. the circular structure of value relations, before. The hierarchy of value priorities, as well as the factor structure of the scale found in our study equally failed to reflect several of the suggested relationships between values, further highlighting age-related deviations from the theoretical structure of value relations. Future research should further examine the theoretical structure of the value system in VOA.

Linear regression showed that only values representing strivings for growth predicted a higher AWB. This is contrary to the idea that in older age, the pursuit of further growth is no longer adaptive due to lacking functional or temporal resources. Rather, while universalism (growth-oriented with social focus) has not been found to be positively related to well-being in some samples of younger persons ([21, 31, 35], or even negatively [23]), it did predict higher AWB in our sample of very old persons. On the other hand, stimulation (growth-oriented, but with personal focus) was unrelated to AWB in our sample, while it was positively related to well-being in other samples [3, 15, 35, 40]. The same applies to achievement, another personally focused value which was positively related to well-being in student samples [31, 35]. Extending previous research on age differences in value priorities, these findings indicate that age not only comes with an increasing concern for one’s human and non-human environment, but that individuals in VOA also profit from it more clearly in terms of their well-being.

For protective goals, in contrast, we found either no significant or a negative relation to AWB. For example, security and tradition, which related negatively to well-being in other samples [3, 21, 35, 40], were unrelated to AWB in our study. It is possible that their social focus outweighs their negative features in VOA; however, what stands out is that they are the most and third most important values respectively in this sample of very old persons, without being positively associated with AWB. In the light of age-related losses in different areas, this supports Bilsky and Schwartz’s [2] assumption that deficiencies increase the importance of goals that would compensate for them. Thus, the values that are most important to an individual are not necessarily those that they benefit from, and people in VOA may be particularly likely to pay less attention to the values that they can actually benefit from. Alternatively, a possible reverse causality can be suggested: people with a higher well-being might have the resources to pursue autonomy and to care for others, whereas individuals with a lower well-being may focus on protective values “whose realization promises relief from anxiety and uncertainty” [35, p. 181].

Relationships between individual values and EwL were similar to those between values and AWB; however, none of the values showed a significant negative relation to EwL. This is in line with the description of valuation of life being mainly predicted by positive features rather than negative ones [20], and indicates that even though the protective features of some individual values lead to a decrease in AWB, they can still provide a purpose for individuals. This could also explain the observed higher level of EwL than AWB and emphasizes the utility of EwL for strength-based interventions of positive psychology [13].

Spirituality was found to be of high relevance to the target group, although less in the sense of institutionalized religion or religious practices. Rather, spirituality of the oldest old predominantly consisted in environmental and interpersonal connectedness as well as a connectedness with God or a higher power. Despite the so-called graying-pattern observed in institutionalized religion and religiosity, this supports previous research on the multidimensionality of spirituality and how it differs from institutionalized religion [16] and highlights that spirituality in a multidimensional understanding that captures more than just religiosity is of importance to people even in VOA [18]. This is further supported by the fact that environmental and interpersonal connectedness were significant predictors of both AWB and EwL. Especially those forms of spirituality offering a sense of communion with the human or non-human environment might thus help to maintain positive affect and purpose in spite of the challenges coming with VOA.

Moreover, institutionalized religion related positively, but religious practices related negatively to EwL. This can be explained by the distinction between extrinsic religiosity, which refers to a social-participative function of religion (such as visiting a mass motivated by a feeling of communion), and intrinsic religiosity, which refers to an individual belief that is embodied in religious practices such as praying [11]. Again, these findings illustrate that it is these aspects of spirituality—representing feelings of community, belonging, and social engagement—which predict high QoL in VOA. This also mirrors our conclusions with respect to the importance of socially focused values in VOA.

All in all, our results confirm that the system of personal goals, beliefs, or strivings can be an important resource, but also a threat for QoL outcomes, as suggested by the CHAPO model of QoL of the very old, although there might also be an interrelation between individual standards and QoL outcomes [43].

## Conclusion

- Individual values are an important element of hedonic as well as eudaimonic QoL in VOA.
- Age-related specifics in the meaning of individual values, their theoretical structure, and their relations to QoL outcomes need to be examined in further research.
- Practitioners should support older people in experiencing maximum autonomy and security in order to enable them to benefit from pursuing further growth.
- A multidimensional and multidirectional concept of spirituality needs to be established in research and recognized as another important element of QoL in VOA.
- Practitioners should be educated in this broad understanding of spirituality in order to support older people on their spiritual journey and development in confrontation with existential questions arising in the face of one’s own finiteness [18].

## Supplementary Information

Appendix A–D: Use of proxy information; control variables; descriptive characteristics based on imputed vs. original dataset; addition to table 2

## References

[1] Asparouhov T, Muthén B (2009). Exploratory structural equation modeling. Struct. Equ. Modeling.

[2] Bilsky W, Schwartz SH (1994). Values and personality. Eur J Pers.

[3] Bobowik M, Basabe N, Páez D, Jiménez A, Ángeles Bilbao M (2011). Personal values and well-being among Europeans, Spanish natives and immigrants to Spain: does the culture matter?. J Happiness Stud.

[4] Boehnke K, Schwartz SH, Stromberg C, Sagiv L (1998). The structure and dynamics of worry: theory, measurement, and cross-national replications. J Pers.

[5] Borg I, Hertel G, Hermann D (2017). Age and personal values: similar value circles with shifting priorities. Psychol Aging.

[6] Datler G, Jagodzinski W, Schmidt P (2013). Two theories on the test bench: Internal and external validity of the theories of Ronald Inglehart and Shalom Schwartz. Soc Sci Res.

[7] Ebner NC, Freund AM, Baltes PB (2006). Developmental changes in personal goal orientation from young to late adulthood: from striving for gains to maintenance and prevention of losses. Psychol Aging.

[8] Erikson E (1980). Identity and the life cycle.

[9] Fegg MJ, Wasner M, Neudert C, Borasio GD (2005). Personal values and individual quality of life in palliative care patients. J. Pain Symptom Manage..

[10] Fisher J (2010). Development and application of a spiritual well-being questionnaire called SHALOM. Religions.

[11] Fuchs B, Bäurle P, Radebold H, Hirsch RD, Studer K, Schmid-Furstoss U, Struwe B (2000). Religiosität und psychische Gesundheit im Alter. Klinische Psychotherapie mit älteren Menschen.

[12] Fung HH, Ho YW, Zhang R, Zhang X, Noels KA, Tam KP (2016). Age differences in personal values: universal or cultural specific?. Psychol Aging.

[13] Gitlin LN, Parisi JP, Huang J, Winter L, Roth DL (2016). Attachment to life: psychometric analyses of the valuation of life scale and differences among older adults. Gerontologist.

[14] Hansen S, Kaspar R, Wagner M, Woopen C, Zank S (2021) The NRW80+ study: conceptual background and design decisions. Z Gerontol Geriat 54. (In press) 10.1007/s00391-021-01970-z PMC8551117 34570267

[15] Haslam N, Whelan J, Bastian B (2009). Big Five traits mediate associations between values and subjective well-being. Pers Individ Dif.

[16] Houtman D, Aupers S (2007). The spiritual turn and the decline of tradition: the spread of post-christian spirituality in 14 western countries, 1981–2000. J Sci Study Relig.

[17] Janhsen A, Golla H, Mantell P, Woopen C (2019). Transforming spirituality through aging: coping and distress in the search for meaning in very old age. J Relig Spiritual Aging.

[18] Janhsen A, Golla H, Romotzky V, Woopen C (2019). Spiritualität im höheren Lebensalter als dynamische Alter(n)saufgabe. Z Gerontol Geriat.

[19] Janhsen A, Woopen C, Hank K, Schulz-Nieswandt F, Wagner M, Zank S (2019). Spiritualität und Alter – Spiritualität im Alter. Handbuch Alternsforschung.

[20] Jopp D, Rott C, Oswald F (2008). Valuation of life in old and very old age: the role of sociodemographic, social, and health resources for positive adaptation. Gerontologist.

[21] Joshanloo M, Ghaedi G (2009). Value priorities as predictors of hedonic and eudaimonic aspects of wellbeing. Pers Individ Dif.

[22] Kantar Public (2018). NRW80+ Methodenbericht.

[23] Karabati S, Cemalcilar Z (2010). Values, materialism, and well-being: a study with Turkish university students. J Econ Psychol.

[24] Kercher K (1992). Assessing subjective well-being in the old-old. The PANAS as a measure of orthogonal dimensions of positive and negative affect. Res Aging.

[25] Kirby SE, Coleman PG, Daley D (2004). Spirituality and well-being in frail and nonfrail older adults. J Gerontol.

[26] Koenig HG (2012). Review article. Religion, spirituality, and health: the research and clinical implications. ISRN Psychiatry.

[27] Lawton MP, Moss MS, Hoffman C, Kleban MH, Ruckdeschel K, Winter L (2001). Valuation of life: a concept and a scale. J Aging Health.

[28] Lifshitz R, Nimrod G, Bachner YG (2019). Spirituality and wellbeing in later life. A multidimensional approach. Aging Ment Health.

[29] Michalos AC (1985). Multiple discrepancies theory. Soc Indic Res.

[30] Neise M, Zank S, Müller SV, Gärtner C (2016). Lebensqualität. Lebensqualität im Alter.

[31] Oishi S, Diener E, Suh E, Lucas RE (1999). Value as a moderator in subjective well-being. J Pers.

[32] Pollack D, Müller O (2013). Religionsmonitor – verstehen was verbindet: Religiosität und Zusammenhalt in Deutschland.

[33] Robinson OC (2013). Values and adult age: findings from two cohorts of the European social survey. Eur J Ageing.

[34] Ryff CD (1989). Happiness is everything, or is it? Explorations on the meaning of psychological well-being. J Pers Soc Psychol.

[35] Sagiv L, Schwartz SH (2000). Value priorities and subjective well-being: direct relations and congruity effects. Eur J Soc Psychol.

[36] Schwartz SH, Zanna M (1992). Universals in the content and structure of values: theory and empirical tests in 20 countries. Advances in experimental social psychology.

[37] Schwartz SH (2003) A proposal for measuring value orientations across nations. http://www.europeansocialsurvey.org/docs/methodology/core_ess_questionnaire/ESS_core_questionnaire_human_values.pdf. Accessed 13 May 2020

[38] Schwartz SH (2012). An overview of the Schwartz theory of basic values. Online Read Psychol Cult.

[39] Schwartz SH, Sortheix FM, Diener E, Oishi S, Tay L (2018). Values and subjective well-being. Handbook of well-being.

[40] Sortheix FM, Lönnqvist JE (2014). Personal value priorities and life satisfaction in europe: the moderating role of socioeconomic development. J Cross Cult Psychol.

[41] Tornstam L (1997). Gerotranscendence: the contemplative dimension of aging. J Aging Stud.

[42] Tulviste T, Kall K, Rämmer A (2017). Value priorities of younger and older adults in seven European countries. Soc Indic Res.

[43] Wagner M, Rietz C, Kaspar R, Janhsen A, Geithner L, Neise M, Kinne-Wall C, Woopen C, Zank S (2018). Quality of life of the very old. Survey on quality of life and subjective wellbeing of the very old in North Rhine-Westphalia (NRW80+). Z Gerontol Geriat.

[44] World Health Organisation (2002) Definition of palliative care. https://www.dgpalliativmedizin.de/images/stories/WHO_Definition_2002_Palliative_Care_englisch-deutsch.pdf. Accessed 30 Nov 2020

