# Supplementary material for: Individual values and spirituality and their meaning for affective well-being and engagement with life in very old age
Source: Z Gerontol Geriatr. 2021 Oct 1;54(Suppl 2):85–92. doi: 10.1007/s00391-021-01974-9 (PMC8551090; doi:10.1007/s00391-021-01974-9)
Supplement: Supplementary file 1 — Appendix A–D: Use of proxy information; control variables; descriptive characteristics based on imputed vs. original dataset; addition to table 2 [file 391_2021_1974_MOESM1_ESM.docx]

***Appendix A.* Use of proxy information.**

It might seem problematic to include proxy information on such subjective positions as were examined in this article. However, proxy reports are an effective and necessary tool to gain representative information on the hard-to-survey very old population, particularly vulnerable subgroups such as those living in institutional settings [2]. Proxy informants were explicitly instructed to choose “don’t know” or “refuse” categories in case they were not sure about the correct answer, which they did more often than target persons [2]. This increases our trust in the available proxy information. In fact, data on individual values and spirituality given by target persons and proxy informants were very similar and showed the same hierarchy of priorities. However, proxy informants were only available for approximately one in eight target persons unable to participate in the study themselves [2], with potential consequences for the representativity of the most vulnerable subgroups.

***Appendix B.* Control variables.**

We considered sociodemographic and health-related variables associated with the dependent variables. Both *age* and *gender* (male/female) were included. *Educational background* was classified as low, medium, or high based on the International Standard Classification of Education [ISCED11, 4]. The *number of treated health conditions* was measured applying an extended version of the Self-administered Comorbidity Questionnaire [1], which presents a list of 19 medical conditions and asks participants whether they are currently treated for them. We used the mean value of all answers as an indicator of *multimorbidity*. Additionally, respondents were asked if they were using *full in-patient care*.

References:

1. Bilsky W, Schwartz SH (1994) Values and personality. Eur J Pers 8:163–181
2. Hansen S, Kaspar R, Wagner M, Woopen C, Zank S (in press) The NRW80+ Study: Conceptual background and design decisions.
3. Sangha O, Stucki G, Liang MH et al (2003) The self-administered comorbidity questionnaire: A new method to assess comorbidity for clinical and health services research. Arthritis Rheum 49:156–163.
4. UNESCO Institute for Statistics (2012) International Standard Classification of Education. ISCED 2011. UIS, Montreal

***Appendix C.* Descriptive characteristics based on the imputed vs. original dataset.**

|  | |  | Imputed dataset |  | Original dataset |
| --- | --- | --- | --- | --- | --- |
|  | |  | M (SD) / % |  | M (SD) / % |
| Age | |  | 85.14 (4.20) |  | 85.14 (4.20) |
| Gender | female |  | 63.7% |  | 63.7% |
|  | male |  | 36.3% |  | 36.3% |
| Education | low |  | 30.6% |  | 30.0% |
|  | middle |  | 51.2% |  | 51.3% |
|  | high |  | 18.2% |  | 18.6% |
| Multimorbidity | |  | 0.18 (0.12) |  | 0.18 (0.12) |
| Full in-patient care | |  | 13.0% |  | 12.7% |
| **Individual values** | |  |  |  |  |
| 1. Security | |  | 3.61 (0.67) |  | 3.61 (0.67) |
| 2. Self-direction | |  | 3.46 (0.70) |  | 3.47 (0.70) |
| 3. Tradition  4. Universalism | |  | 3.30 (0.88)  3.20 (0.90) |  | 3.31 (0.87)  3.22 (0.90) |
| 5. Hedonism | |  | 2.97 (0.89) |  | 2.98 (0.89) |
| 6. Conformity | |  | 2.94 (1.03) |  | 2.94 (1.03) |
| 7. Benevolence | |  | 2.76 (0.96) |  | 2.77 (0.97) |
| 8. Achievement | |  | 2.60 (0.94) |  | 2.62 (0.94) |
| 9. Power | |  | 1.94 (0.88) |  | 1.94 (0.88) |
| 10. Stimulation | |  | 1.57 (0.80) |  | 1.57 (0.80) |
| **Spirituality** | |  |  |  |  |
| Transcendental connectedness | |  | 2.91 (1.05) |  | 2.93 (1.05) |
| Interpersonal connectedness | |  | 3.44 (0.69) |  | 3.44 (0.69) |
| Environmental connectedness | |  | 3.47 (0.73) |  | 3.48 (0.73) |
| Institutionalized religion | |  | 2.39 (1.17) |  | 2.39 (1.17) |
| Feeling part of a greater whole | |  | 2.56 (1.04) |  | 2.57 (1.06) |
| Religious practices | |  | 2.61 (1.20) |  | 2.61 (1.20) |
| Faith or spirituality in life | |  | 2.80 (1.11) |  | 2.80 (1.11) |
| **QoL outcomes** | |  |  |  |  |
| Affective well-being | |  | 3.25 (0.89) |  | 3.25 (0.89) |
| Engagement with life | |  | 1.45 (0.62) |  | 1.44 (0.62) |

***Appendix D.* Addition to table 2: Differing results of hierarchical regression models predicting affective well-being and engagement with life by spirituality and individual values based on the original dataset (n=1863)**

In the original data, some different results occurred: in AWB (III), age (-.05*) was significant, full in-patient care and connection to men weren’t. In EwL (I), gender (-.06*) was significant. In EwL (II), low education and institutionalized religion weren’t significant. In EwL (III), connection to men, institutionalized religion and hedonism weren’t significant. *p≤0.05 **p≤0.01 ***p≤0.001
